# Promising Preventive Strategies for Intraventricular Hemorrhage in Preterm Neonates: A Critical Review

**DOI:** 10.3390/jcm14196763

**Published:** 2025-09-24

**Authors:** Niki Dermitzaki, Maria Baltogianni, Chrysanthi Maria Tsiogka, Aikaterini Nikolaou, Foteini Balomenou, Vasileios Giapros

**Affiliations:** Neonatal Intensive Care Unit, School of Medicine, University of Ioannina, 45500 Ioannina, Greece; n.dermitzaki@uoi.gr (N.D.); mbalt@doctors.org.uk (M.B.); chrisatsiogka@gmail.com (C.M.T.); nikaikaterini@gmail.com (A.N.); f.balomenou@uoi.gr (F.B.)

**Keywords:** intraventricular hemorrhage, preterm neonates, prevention, prevention bundles, indomethacin, erythropoietin, insulin-growth factor-1, stem cells

## Abstract

Intraventricular hemorrhage (IVH) is a common complication of prematurity and continues to represent a considerable threat due to its association with significant short- and long-term morbidity and mortality. Despite the advances in neonatal care, the prevalence of IVH, particularly in the extremely preterm neonates, remains high. Therefore, it is imperative to recognize and implement in clinical practice preventive strategies, non-pharmacological or pharmacological, to reduce IVH effectively. The aim of this narrative review is to provide an overview of novel and debatable preventive measures for IVH that are promising for clinical use and could potentially improve outcomes for very preterm neonates. IVH prevention bundles (IVHPBs) consist of strategies that aim to minimize hemodynamic and cerebral perfusion fluctuations, which are a crucial component of IVH pathogenesis. Early postnatal prophylactic indomethacin, erythropoietin, and insulin-growth factor-1 administration have shown encouraging results on IVH prevention; however, the literature is still inconclusive. Stem-cell-based interventions represent novel and promising techniques with the potential to contribute to the prevention of IVH. The prevention of IVH remains a field of investigation, and there is a requirement for conclusive evidence and recommendations. The necessity for further large-scale prospective studies is therefore evident.

## 1. Introduction

Despite the significant advances in perinatal care, prematurity remains a leading cause of neonatal morbidity and mortality [1,2]. Intraventricular hemorrhage (IVH) is a major contributor to preterm brain injury and is associated with adverse neurodevelopmental outcomes. It is an early complication of prematurity that typically occurs within the first three postnatal days, mainly affecting neonates with a gestational age (GA) of less than 28 weeks [2]. The incidence of IVH is inversely correlated with GA, and differs among centers and geographic regions [3]. Reported rates are up to 40% in neonates with a GA of less than 28 weeks, and 27% in neonates born before 32 weeks of gestation [2,4,5]. Despite the considerable improvement that has been achieved in the survival rates of preterm neonates, there is an inconsistency in the trend of IVH rates over time among the different studies. A number of studies have reported a decline in IVH rates compared to previous decades; however, others have reported no difference [2,6,7,8]. An Australian study compared the incidence of IVH rates in a large cohort of preterm neonates with a GA < 32 weeks in 17 years, and observed a significant decline in both IVH of any grade (23.6% vs. 21.4%, *p* < 0.01) and severe IVH (4% vs. 2.8%, *p* < 0.01) [6]. However, a recent systematic review and meta-analysis compared the incidence of IVH in studies conducted prior to and following 2007, and reported no significant difference [2].

IVH typically originates from the periventricular germinal matrix (GM) located within the caudothalamic groove, underneath the ventricular ependyma. The GM is a highly cellular, vascularized, and metabolically active region. It is characterized by an irregular, immature capillary network that lacks sufficient structural support. GM begins to involute after 24 weeks of gestation and is typically absent in neonates born at term [9,10]. Moreover, this fragile network is located at the border between the small cerebral arteries and the deep cerebral vein system, rendering it vulnerable to hemodynamic fluctuations [11].

**Figure 1:**
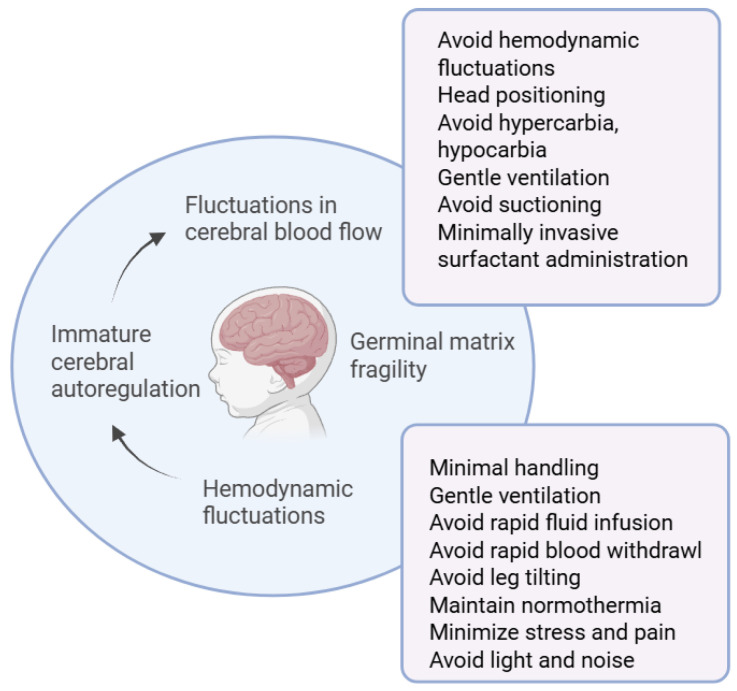
Pathogenesis of IVH and preventive strategies.

The pathogenesis of IVH is multifactorial and is mainly attributed to the fragility of the GM and fluctuations in cerebral blood flow. Impaired cerebral autoregulation in preterm neonates, particularly those who are clinically unstable, leads to pressure-passive cerebral blood flow, which is the inability to maintain constant blood flow in response to fluctuations in arterial pressure (Figure 1). Hypercarbia, acidemia, anemia, and hypoglycemia have also been associated with cerebral blood flow fluctuations. Cerebral blood flow fluctuations increase the likelihood of vascular rupture in the GM. The subsequent hemorrhage may be contained within the GM or extend to the lateral ventricle [10,11].

The severity of IVH is graded based on the extent and presence of dilatation of the lateral ventricle, primarily using the Papile or Volpe grading systems [12,13]. Severe intraventricular hemorrhage (sIVH) refers to grades III and IV hemorrhages, which are characterized by ventricular dilatation and/or extension into the parenchyma [10].

Severe IVH is associated with increased mortality and adverse neurodevelopmental outcomes in survivors, such as intellectual disability, motor deficits, hearing and visual impairment, and cerebral palsy [14,15]. A recent prospective cohort study reported mortality rates of 55.2% and neurodevelopmental impairment at the corrected age of 18–24 months in 43.3% of infants with sIVH [15]. Moreover, a recent meta-analysis has reported a three- to four-fold increased risk of moderate to severe neurodevelopmental impairment at the age of three years in neonates with sIVH [16]. Conversely, data on the neurodevelopmental outcomes of infants with low-grade IVH are still inconclusive and remain a topic of debate [15,17,18,19,20,21,22]. Several studies have observed a comparable neurodevelopmental outcome between infants with low-grade IVH and those with no IVH [15,17,18,19]. However, two recent meta-analyses concluded that infants with low-grade IVH are at an increased risk of neurodevelopmental impairment compared to infants without IVH [14,16].

The potential detrimental effects of IVH on the survival and neurodevelopment of preterm neonates underscore the necessity for the implementation of effective preventative strategies to reduce the incidence of IVH in this vulnerable population. The primary antenatal preventive measures for IVH consist of antenatal corticosteroids and magnesium sulfate administration, antenatal transfer and delivery at centers with expertise in neonatal care, and the still debatable cesarean section in periviable births [23,24,25,26,27,28]. Delayed cord clamping and advances in neonatal care, including non-invasive respiratory support, surfactant administration, and circulatory management, are some of the perinatal strategies that contribute to the prevention of IVH [6,11,29,30,31].

Although several antenatal and perinatal strategies have been proven to be effective in the prevention of IVH and have been widely implemented, the incidence of IVH among preterm neonates remains high. Therefore, in view of the increasing survival of the most vulnerable extremely preterm neonates, there is a necessity for more effective strategies to achieve a decline in IVH rates and to improve both survival and long-term prognosis of these neonates. This narrative review aims to provide clinicians with a critical overview of the current literature on novel and debatable preventive measures for IVH that are promising for clinical use and could potentially improve outcomes for very preterm neonates.

## 2. Methods

**Figure 2:**
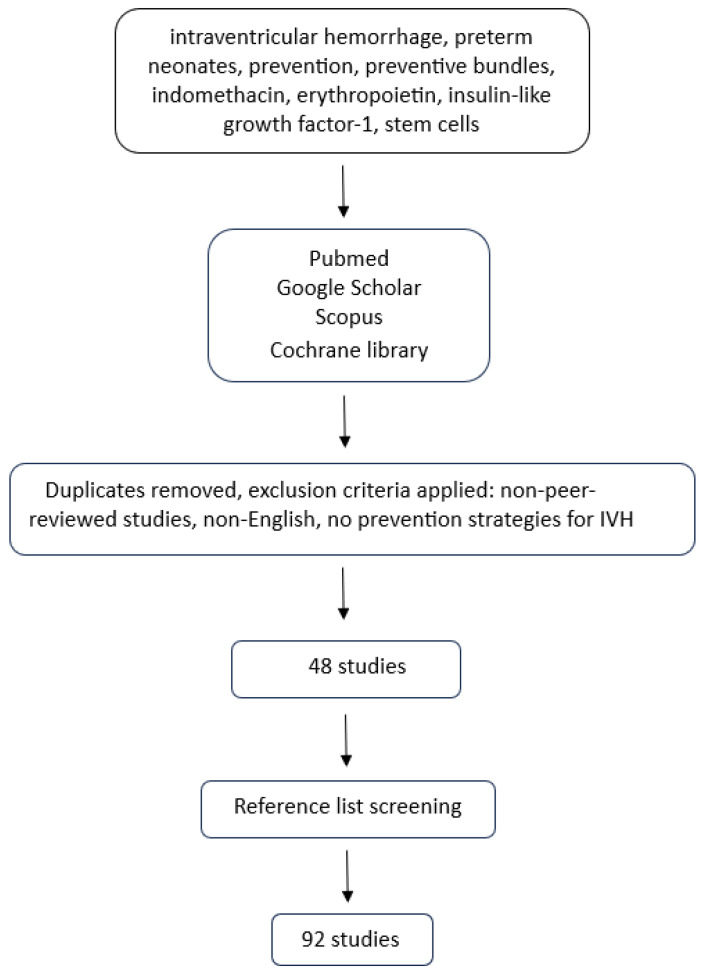
Flow diagram of the study selection process.

This review was conducted in accordance with the Scale for the Assessment of Narrative Review Articles (SANRA), aiming to ensure methodological rigor and quality. To ensure an extensive analysis of the research topic, a structured narrative review checklist (SANRA checklist) was utilized (Supplementary Table S1). A systematic review or meta-analysis was not feasible due to significant heterogeneity in the methodology, populations, interventions, and outcomes of existing studies evaluating preventive measures for IVH in preterm neonates. A narrative review was therefore considered the most appropriate approach for critically synthesizing and analyzing the current literature. To identify relevant studies a structured and comprehensive search was conducted using online databases: PubMed, Scopus, Cochrane Library, and Google Scholar databases from inception up to June 2025. The following string was used in the Pubmed: *“intraventricular hemorrhage” OR “IVH” AND “preterm neonates” OR “premature infants” AND “prevention” AND “preventive bundles” OR “care bundles” OR “indomethacin” OR “erythropoietin” OR “EPO” OR “insulin-like growth factor 1” OR “IGF-1” OR “stem cells”* and was adapted for search in the other databases. Meta-analyses, systematic reviews, randomized control trials (RCTs), retrospective and prospective cohort studies were included. Studies not published in English, non-peer-reviewed studies, and studies not evaluating preventive strategies for IVH were excluded from the analysis. Furthermore, the reference lists of all retrieved articles were screened to identify any additional relevant studies that may not have been initially identified in the search (Figure 2).

The selection of articles and the extraction of data were conducted independently by two authors. This process involved screening the titles and abstracts of articles, followed by a full-text evaluation. In instances of uncertainty, the decision-making process involved discussions with the co-authors. Data extraction from all included studies was performed using a standardized approach to evaluate and compare the literature. The following variables were extracted: study design, sample size and population characteristics, control group, intervention, outcomes, main findings, and reported limitations. Due to the narrative nature of the review, a formal tool for risk of bias assessment was not used, which represents a limitation as it prevents a fully systematic evaluation of the included studies. However, potential biases and methodological limitations of the included studies were evaluated.

## 3. Results

### 3.1. IVH Prevention Bundles (IVHPB)

**Figure 3:**
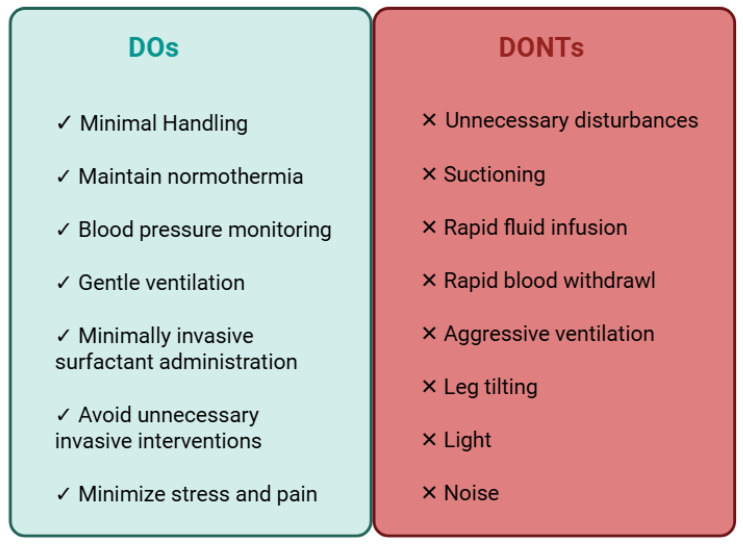
Routine care strategies for preterm neonates during the first 72–96 h after birth to prevent fluctuations in blood pressure and cerebral blood flow.

IVH prevention bundles (IVHPB) aim to minimize hemodynamic and cerebral perfusion fluctuations during the critical period of the first postnatal days. In an aim to achieve this objective, a number of strategies are employed as part of the routine care of preterm neonates. These include minimal handling, head positioning, blood pressure management, thermoregulation, appropriate respiratory support, the avoidance of rapid intravenous fluid administration or blood draws, and the avoidance of stress and pain (Figure 3) [32,33,34]. The common rationale of all these diverse strategies is to prevent hemodynamic fluctuations.

Minimal handling is a broad term that encompasses all practices aimed at minimizing disturbance during the routine care of preterm neonates. It has been demonstrated that routine procedures are associated with significant circulatory alterations in this vulnerable population [35]. An aspect of minimal handling involves adhering to specified care times and clustered care, thereby avoiding unnecessary disturbances and maximizing rest periods, while employing two-person handling during interventions and care [36]. Although controversial, it has been suggested that tilting the head and avoiding rotation may facilitate cerebral blood drainage and therefore decrease the risk for IVH [34]. Both rapid infusion of fluids and rapid withdrawal of blood can alter cerebral hemodynamics due to increased cerebral perfusion and decreased cerebral oxygenation, respectively, and should therefore be avoided [37]. Aspiration of the endotracheal tube, a routine procedure for intubated neonates, should be minimized during the first postnatal days, as it has been associated with alterations in cerebral blood flow [34]. Non-invasive respiratory support and minimally invasive techniques for surfactant administration should be used instead of intubation and mechanical ventilation, provided it is feasible [38]. Moreover, leg tilting should be avoided, as it has been shown to significantly reduce cerebral oxygenation due to an abrupt increase in venous return [35,37]. Painful procedures, loud noises, and light should be avoided, as these have been associated with alterations in cerebral hemodynamics [35,39,40]. Maintaining normothermia (between 36.5 °C and 37.5 °C) during the initial postnatal period is crucial. It has been established that both hypothermia and hyperthermia have the potential to induce fluctuations in blood pressure and metabolic disturbances, which can consequently result in cerebral blood flow fluctuations [41]. The association of both conditions with IVH or perinatal brain injury has been suggested [42]. However, despite the physiological rationale of all these strategies, inconsistent results regarding the efficacy of IVHPB as a preventive measure to decrease sIVH incidence in preterm neonates have been reported in different studies (Table 1) [32].

A significant reduction in the incidence of IVH of any grade in preterm neonates with a GA < 30 weeks was reported by de Bijl-Marcus et al., after the implementation of a series of nursing interventions, including the following: the maintenance of a tilted midline head position; the avoidance of elevation of the legs; and the avoidance of rapid fluid flushing or blood withdrawal during the first 72 postnatal hours. The preventive efficacy of these interventions was more notable in the group of neonates with lower gestational ages (GA < 27 weeks). This is probably due to the greater impact of these preventive strategies on the most immature and vulnerable populations [43]. Wong et al. observed a significant reduction in IVH rates following the implementation of neuroprotective strategies, including positioning and minimal handling, in a population of preterm neonates with a GA of less than 26 weeks [44]. A recent study also reported a significant reduction in the incidence of IVH (24.4% vs. 9.3%) after the introduction of strategies to minimize cerebral blood flow fluctuations in the routine care of preterm neonates (GA < 30 weeks) during the first three days of life [34].

Nevertheless, a number of other studies have reported failure of the IVHPB to reduce the rate of IVH in preterm populations [45,46]. In addition to differences in study populations, poor adherence to IVHPB may explain the conflicting observations regarding the efficacy in preventing IVH among different studies. This was demonstrated by Kolnik et al., who reported a significant reduction in the rates of IVH with improved adherence to clinical protocols. The incidence of sIVH in preterm neonates was compared between two epochs, when adherence to IVHPB increased from 24% (pre-intervention period) to 88% (intervention period), resulting in a 76% reduction in sIVH (9.8% vs. 2.4%) [32]. Tang et al. also observed a significant decrease in sIVH rates following the introduction of a bedside assessment tool that provided medical staff with real-time assessments of care bundle implementation and immediate feedback [33]. However, a recent, large, systematic review concludes that the reasons for the inconsistency in the efficacy of clinical strategies implemented to prevent IVH in different settings are not known [47].

**Table 1:** Studies evaluating the efficacy of intraventricular hemorrhage prevention bundles in the reduction in IVH.

Author	Study Design	Population	Groups	Intervention	Outcome Measures	Main Findings of Interest	Limitations
de Bijl-Marcus, 2020 [43]	Multicenter cohort	561 neonates, GA < 30 weeks	281 intervention group 280 pre-intervention	Positioning (tilted MHP) Avoidance of rapid fluid infusion or blood withdrawal Avoidance of leg elevation	IVH rate cPVL in-hospital mortality	IVH any grade, cPVL and/or mortality: OR 0.42, 95% CI 0.27–0.65 sIVH, cPVL, and/or mortality: OR 0.54, 95% CI 0.33–0.91	Not randomized The recruitment period was different in each center. The effectiveness of each intervention could not be assessed. Potential variation in reporting of cUS and determining the grade of the IVH
Persad, 2021 [46]	Single-center retrospective cohort	404 neonates, GA < 30 weeks	215 intervention group 189 pre-intervention	Optimization of antenatal care Positioning (tilted MHP) Minimal handling	IVH rate	sIVH: 9.8% vs. 6.9%, *p* = 0.37	The effectiveness of each intervention could not be assessed. No monitoring of adherence Potential variation in reporting of cUS and determining the grade of the IVH.
Gross, 2021 [45]	Single-center retrospective cohort	229 neonates, GA < 30 weeks or BW< 1250 g	107 intervention group 122 pre-intervention	Positioning (tilted MHP) Minimal handling	IVH rate	IVH any grade: OR 1.02; 95% CI 0.57–1.84 sIVH: OR 1.0; 95% CI 0.67–1.55	Single-center, small sample Not randomized CUS on the fourth day
Kolnik, 2023 [32]	Single-center quality-improvement	425 neonates, GA < 30 weeks or BW< 1250 g	185 intervention group 240 pre-intervention	Improvement of adherence to IVHPB (positioning, minimal handling, thermoregulation)	IVH rate	IVH any grade: OR 0.30; 95% CI 0.10–0.90	Single-center No monitoring of adherence Severity of illness bias (reduced adherence in critically ill)
Tang, 2025 [33]	Single-center quality-improvement	86 neonates, GA < 26 weeks	21 intervention group 65 pre-intervention	Improvement of adherence to IVHPB	IVH rate	sIVH: 18.8% vs. 39.2%, *p* = 0.14	Single-center, small sample No monitoring of adherence
Peltola, 2025 [34]	Single-center quality-improvement	122 neonates, GA < 30 weeks	78 intervention group 44 pre-intervention	Positioning (tilted MHP) Minimal handling	IVH rate	IVH any grade: 9.3% vs. 24.4%	Limited intervention period

GA: gestational age; MHP: midline head position; IVHPB: intraventricular hemorrhage prevention bundles; sIVH: severe intraventricular hemorrhage; BW: birthweight; OR: odds ratio; CI: confidence interval.

### 3.2. Head Position

Fluctuations in cerebral blood flow, in association with impaired autoregulation, contribute to the pathogenesis of IVH in preterm neonates. It has been proposed that the position of the head can affect cerebral hemodynamics and potentially impact the development of IVH during the critical first days of life in the most vulnerable population [37]. The optimal posture for preterm neonates to avoid cerebral blood flow fluctuations has been the subject of investigation for many years. Although elevated midline head positioning (MHP) is widely adopted during the early transitional period in clinical practice and is a core part of IVHPB, there is still a lack of conclusive evidence on its effectiveness in preventing IVH [48].

As the two internal jugular veins represent the main outflow path for cerebral blood, it has been suggested that the lateral position of the head is associated with compression of the homolateral jugular vein, which can compromise venous return and lead to venous congestion and increased intracranial pressure and cerebral blood flow [49,50,51]. Conversely, it has been demonstrated that an elevated head position is associated with reduced central venous pressure and facilitates cerebral venous drainage [52].

A discrepancy exists among studies investigating the effect of different head positions on cerebral hemodynamics and oxygenation in neonates [53]. Ancora et al. evaluated brain hemodynamics in 24 clinically stable, very preterm neonates at six different positions using near-infrared spectroscopy (NIRS) and concluded that the effects depend on GA. No significant differences were observed in tissue oxygenation or tissue hemoglobin index. However, a decrease in cerebral blood volume was noted following head rotation in the subgroup of neonates with a GA of less than 26 weeks, as indicated by a decrease in the tissue hemoglobin index. The tissue oxygenation index remained stable [54]. Pellicer et al. conducted a study on a cohort comprising both preterm and term neonates, reporting a significant increase in cerebral blood volume with lateral head position [55]. A subsequent study by Liao et al. evaluated the effect of short-term head position changes on regional cerebral saturations in a cohort of 20 relatively stable preterm neonates with a mean GA of 26^+5^ weeks during the first three postnatal days. Although a significant decrease in regional cerebral saturation on the ipsilateral side when the head was turned to the left lateral was noted, this was determined as probably clinically insignificant. The authors concluded that brief postural changes in the head did not affect cerebral oxygenation in stable preterm neonates [53]. In a more recent study, stable very preterm neonates did not have altered cerebral blood flow velocities or regional cerebral oxygenation when the posture changed from supine MHP to prone with a lateral head position [56]. A systematic review concluded that data regarding the effects of head posturing on cerebral hemodynamics and oxygenation in preterm neonates are inconclusive [37].

In an RCT involving 48 preterm neonates with a GA of less than 30 weeks, Al-Abdi et al. randomized the subjects to be cared in either the MHP or lateral head position (both groups in 0° tilt) for the first postnatal week, and reported no difference in the incidence of IVH [57]. A multicenter RCT from the same group of authors was terminated early due to a low recruitment rate. However, no difference in the incidence of IVH was observed between the MHP and lateral head position groups in the 72 neonates enrolled [58]. Kochan et al. randomized 180 extremely low-birth-weight neonates (ELBW) to be placed in either an elevated MHP (30° tilt) or a flat supine position for the first four postnatal days. The overall incidence of IVH was similar in the two groups; however, the incidence of IVH grade IV was significantly lower in the MHP group. Interestingly, although no difference in the incidence of common neonatal morbidities related to preterm birth was observed between the two groups, the in-hospital survival rate was significantly higher for neonates in the MHP group (88% vs. 76%) [59]. However, in a large multicenter study involving 2021 very-low-birth-weight (VLBW) neonates, the incidence of IVH was comparable between the elevated MHP and routine posture groups, but the risk for developing sIVH was higher in the MHP group. The authors hypothesized that this increased risk may be attributable to agitation in the neonates when maintained in a fixed position for a protracted period [60]. A recent meta-analysis concluded that, according to low-quality data, there is no impact of MHP on the incidence of IVH [61].

In conclusion, although elevated MHP is incorporated into prevention bundles for IVH and is part of routine clinical practice for caring for preterm neonates during the first postnatal days in many NICUs based on physiological rationale, definitive evidence of the effectiveness of this practice is lacking. The presence of inconsistency among studies and methodological limitations precludes the establishment of definitive conclusions. It is imperative to consider the results of the multicenter study by Kumar et al., which demonstrated an elevated risk of sIVH in the MHP group. This underscores the necessity for additional data on the implementation of MHP in routine clinical care before any recommendations can be made [60]. High-quality evidence from well-designed, large RCTs is needed [48].

### 3.3. Minimally Invasive Surfactant Administration

Respiratory support and the administration of surfactant are integral to the care of preterm neonates in the first postnatal period [62]. Respiratory distress syndrome (RDS) is a significant cause of morbidity in preterm neonates, with an incidence that inversely correlates with gestational age [63]. Non-invasive respiratory support and minimally invasive surfactant administration have been associated with reduced neonatal morbidities and are recommended in current guidelines [63].

The impaired autoregulation of cerebral blood flow in preterm neonates increases their vulnerability to alterations in partial pressure of blood gases and arterial blood pressure. Intubation and surfactant administration through the intubation–surfactant–extubation (INSURE) method are associated with hemodynamic and blood gases fluctuations. It has been suggested that minimally invasive surfactant administration, characterized by reduced fluctuations in blood pressure and more stable arterial CO_2_, may be associated with a reduced risk of IVH [64,65,66].

A number of studies have reported an association between minimally invasive surfactant administration and a reduced incidence of IVH in preterm neonates. In a prospective cohort study, Pérez-Iranzo et al. compared the incidence of sIVH in 108 preterm neonates who received surfactant through less invasive surfactant administration (LISA) with a historical control group of 100 neonates who received surfactant with the INSURE method. A significantly reduced incidence of sIVH was reported in the LISA group [odds ratio (OR): 0.054, 95% Confidence Interval (CI) 0.01–0.2] [67]. In an RCT, Kribs et al. also reported significantly lower rates of sIVH in preterm neonates with a GA of less than 27 weeks who received surfactant via LISA compared to those who received it by intubation (10.3% vs. 22.1%, *p* = 0.02) [68]. A recent Cochrane meta-analysis reported a low level of certainty that the incidence of sIVH in neonates who received surfactant through a thin catheter was significantly reduced, in comparison with those who received it through an endotracheal tube [risk ratio (RR) 0.63, 95% CI 0.42–0.96] [69]. However, the existing literature is inconsistent. A previous multicenter RCT reported no significant difference in the incidence of sIVH in neonates who received surfactant with a thin catheter compared to standard care (7% vs. 5%, *p* = 0.59) (409). Moreover, Aldana-Aguirre et al., in a systematic review of six RCTs, observed no significant effect on IVH rates (RR 0.69, 95% CI 0.40–1.17) [70].

It has been demonstrated that surfactant administration in spontaneous breathing neonates with a thin catheter is associated with less hemodynamic fluctuations and more stable blood gases. Based on this rationale, it has the potential to minimize the risk of IVH. However, evidence in the existing literature is not conclusive, and future large prospective studies are needed to confirm this association.

### 3.4. Indomethacin

Indomethacin, a cyclooxygenase inhibitor that has been used in NICUs since the 1970s primarily for ductal closure, remains a common medication for preterm neonates and has been ranked as the tenth most commonly used drug for ELBW neonates [71,72]. Indomethacin inhibits prostaglandin synthesis through inhibition of cyclooxygenase pathways. Moreover, it suppresses the hyperemic responses of the cerebral vascular network in situations of hypercapnia and hypoxia, and prevents cerebral ischemia due to impaired perfusion [73,74,75]. Indomethacin has also been demonstrated to promote basement membrane deposition in germinal matrix microvessels [76].

**Figure 4:**
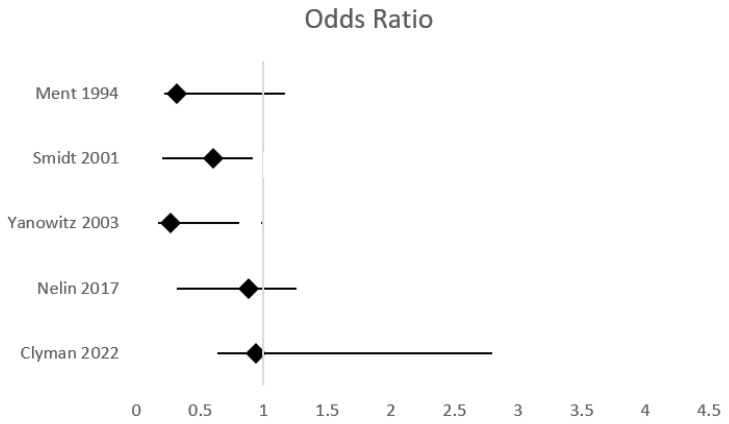
Effect of indomethacin versus control on severe IVH incidence.

Several studies conducted in the 1990s, including two large RCTs, suggested that the early postnatal administration of a low dose of indomethacin as prophylaxis is associated with a reduced risk of IVH development [77,78,79] (Table 2, Figure 4). Several recent studies have also demonstrated the benefits of indomethacin in IVH prevention [80,81,82,83]. Indomethacin prophylaxis was associated with a significant reduction, compared to no prophylaxis, of IVH of any grade (OR 0.47, 95% CI 0.27–0.79), sIVH (OR 0.24, 95% CI: 0.09–0.61), and mortality (OR 0.3 (95% CI: 0.14–0.65) in a large cohort of preterm neonates with a GA < 26 weeks born in the context of amniotic infection syndrome [80]. A meta-analysis of 15 trials demonstrated, with moderate certainty, a moderate reduction in sIVH with indomethacin prophylaxis (RR 0.64, 95% CI, 0.52–0.79) [84]. The recently updated Cochrane meta-analysis on this topic concluded with moderate certainty that prophylactic indomethacin is associated with a small reduction in sIVH (network RR 0.66, 95% CI 0.49–0.87) and a moderate reduction in mortality (network RR 0.85, 95% CI 0.64–1.1) [85].

However, several recent studies have not proven that early indomethacin administration reduces the incidence of IVH, possibly due to significant changes in antenatal and perinatal medical practice since the two large RCTs [86,87,88]. Recently, Clyman et al. reported no significant difference in the rate of IVH (OR 0.94, 95% Cl 0.30–2.92) and other neonatal morbidities among neonates with a GA of less than 25 weeks when different epochs at which indomethacin prophylaxis was routinely administered were compared with an epoch at which it was not administered [87]. Szakmar et al. also reported no reduction in the incidence of sIVH in extremely preterm neonates in the period following the implementation of a prophylactic indomethacin protocol [88]. In a combined meta-analysis of RCTs and retrospective cohort studies, Al-Matary et al. reported no significant difference in the incidence of sIVH between neonates who received prophylactic indomethacin and those who did not (OR 1.03; 95% CI 0.67–1.57) [89].

Several researchers have hypothesized that the benefits of prophylactic indomethacin may be more significant for preterm neonates at high risk of sIVH, and have investigated the effectiveness of risk assessment and targeted prophylaxis [81,90,91,92]. Luque et al. demonstrated that indomethacin prophylaxis is associated with a lower risk of IVH in a cohort of preterm neonates with a birthweight of less than 1250 g. The benefit was found to be more substantial among neonates at a higher risk of sIVH [81]. A recent multicenter retrospective study by Chawla et al. investigated the potential benefits of targeted prophylactic indomethacin administration to a group of extremely preterm neonates who were at a higher risk of IVH according to a risk prediction model based on clinical variables. The study demonstrated no reduction in the incidence of sIVH [92]. The study conducted by Foglia et al. suggests that the relative treatment effect on the risk of sIVH development showed no significant variation across the different groups, regardless of their baseline risk of IVH development. However, the absolute treatment effect was found to be dependent on the baseline risk of sIVH in the population, corresponding to a number needed to treat (NNT) of 71 in the low-risk group and 11 in the high-risk group to prevent sIVH [93].

The proposed dosing regimen based on the two early RCTs was 0.1 mg/kg/day intravenously for three days, starting 6–12 h after birth [77,78]. However, a recent retrospective study demonstrated that a single dose of indomethacin (0.2 mg/kg) within the first six hours was non-inferior to the standard regimen in terms of rates of brain injury [94]. Gillam-Krakauer et al. retrospectively compared neonates with a GA of less than 29 weeks who received a single dose of indomethacin (0.2 mg/kg) at birth with those who did not. The GA-adjusted incidence of IVH was lower in the treated group (OR 0.58, 95%CI 0.36, 0.94) [82]. In two retrospective studies, Mizra et al. compared the efficacy of prophylactic indomethacin initiated prior to 6 h of age and between 6 and 12 h, reporting similar efficacy in both groups [95,96].

Despite the potential for a reduced rate of sIVH in preterm neonates, a number of longitudinal studies have not demonstrated a benefit of prophylactic indomethacin at birth in terms of survival and neurodevelopmental outcome. Ment et al. compared the long-term neurodevelopmental outcomes at 36 months and 4.5 years of age in preterm neonates with a birth weight of less than 1250 g who received prophylactic indomethacin within 12 h of birth, and in neonates who received a placebo. They reported no significant difference in the rates of cerebral palsy or neurosensory impairment. A lower incidence of intellectual disability was observed among children who received prophylactic indomethacin at birth at the age of 4.5 years, which was not sustained at the age of 12 years [97,98]. A large multicenter RCT (Trial of Indomethacin Prophylaxis in Preterm—TIPP) by Schmidt et al., involving 1202 ELBW neonates, reported a significant reduction in the incidence of sIVH among neonates treated with prophylactic indomethacin compared to placebo. However, no benefit of the composite outcome of death or severe neurodevelopmental impairment at the age of 18 months was demonstrated [77]. In a post hoc analysis of the TIPP trial, Foglia et al. recently investigated the long-term effects of targeted indomethacin prophylaxis on ELBW neonates at a higher risk of sIVH and concluded that selective prophylaxis did not offer an advantage in terms of survival or neurodevelopmental outcome [93]. A Cochrane meta-analysis reported no effect on the composite outcome of mortality and neurodevelopmental impairment at corrected age of 18 to 36 months in treated neonates (typical RR 1.02, 95% CI 0.90–1.15) [99].

Indomethacin has vasoconstrictive properties, which may consequently alter blood flow in the central nervous, renal, and gastrointestinal systems [100]. Therefore, concerns have been raised about the potential adverse effects of indomethacin use in preterm neonates, including spontaneous intestinal perforation (SIP), necrotizing enterocolitis (NEC), impaired renal function, and ischemic brain injury [85]. Despite the observed reduction in cerebral blood flow, indomethacin prophylaxis has not been associated with impaired neurodevelopment outcome in several studies [77,101]. Moreover, a recent Cochrane review revealed no significant differences in the incidence of cerebral palsy between neonates who received indomethacin prophylaxis and those who received a placebo (network RR 0.97, 95% CI 0.44 to 2.1) [85]. The association between early prophylactic indomethacin and SIP is the most extensively studied. It has been hypothesized that a number of factors may contribute to the pathogenesis of SIP in relation to indomethacin use. These include reduced mesenterial blood flow, altered mucosal permeability, and increased reactive oxygen species [102]. However, the literature is conflicting [101]. Several studies have demonstrated an increased risk of SIP with early prophylactic indomethacin [102,103,104]. In contrast, other studies have not reported an association [77,82,83]. A recent Cochrane review reported a negligible difference in the incidence of SIP (network RR 0.92, 95% CI 0.11–3.9) with moderate certainty and in NEC (network RR 0.92, 95% CI 0.11–3.9) with high certainty, between neonates who received prophylactic indomethacin compared to those who received a placebo [85]. A common practice during indomethacin administration is to withhold enteral feeding to avoid a potential surcharge in the gastrointestinal system [102]. However, a large population-based Canadian study demonstrated that the risk of SIP in preterm neonates of a GA less than 30 weeks treated with indomethacin was independent of enteral feeding [102]. Moreover, Kelleher et al. also found no difference between enterally fed and non-fed ELBW neonates exposed to early prophylactic indomethacin (RR 0.74, 95% CI 0.49–1.11) [105]. Another concern regarding the use of indomethacin prophylaxis in preterm neonates is the potential renal function impairment due to decreased renal perfusion [106]. A significant increase in serum creatinine and a decrease in glomerular filtration rate (GFR) were demonstrated by Akira et al. in a retrospective cohort of neonates with a GA of less than 30 weeks, during the first days of indomethacin administration, in comparison with a control group of neonates not exposed to indomethacin. However, renal function returned to normal by day 30 [107]. Other studies have also reported transient elevations in serum creatinine or oliguria in treated preterm neonates [77,108,109]. Despite the necessity for additional data to reach conclusive evidence, close monitoring of renal function is recommended [108].

The lack of an established long-term benefit of early indomethacin prophylaxis, coupled with concerns regarding potential adverse effects, has resulted in a diversity of practices among NICUs [92]. It has been hypothesized that the potential benefits may outweigh the risks in preterm neonates at a higher risk of IVH, and a risk-based prophylaxis has been proposed rather than routine administration [102,110]. A recent consensus has proposed the use of early prophylactic indomethacin in preterm neonates with a GA less than 25 weeks and neonates with a GA 25 to 27^+6^ weeks and postnatal age less than 72 h that remained intubated or were intubated beyond 24 h [110]. Nonetheless, in the absence of established guidelines, considerable heterogeneity in clinical practices is observed across different centers.

**Table 2:** Studies investigating the efficacy of indomethacin prophylaxis for preventing IVH compared with routine care.

Author	Type of Study	Population	Study Groups	Intervention	Objective	Outcome of Interest	NNT	Main Conclusions	Limitations
Ment, 1994 [78]	RCT	431 neonates, BW 600–1250 g	209 IP-222 placebo	0.1 mg/kg at 6–12 h, followed by 0.1 mg/kg/day for 2 days	Incidence of IVH	sIVH: RR 0.32, 95% CI 0.099–1.1	IVH any grade: 25 sIVH: 25	IP was associated with reduced rate of IVH and particularly grade IV IVH	Relatively small sample size
Smidt, 2001 [77]	RCT	1202 neonates, BW 500–999 g	601 IP-601 placebo	0.1 mg/kg/day for 3 days	Mortality or NDI at CA 18 months Incidence of IVH and other preterm morbidities	IVH any grade: OR 1.0, 95% CI 0.8–1.3 sIVH: OR 0.6, 95% CI 0.4–0.9	25	IP reduced the rate of sIVH and PDA IP did not improve survival without neurosensory impairment at 18 months	Neonates with preexisting IVH were not excluded
Yanowitz, 2003 [83]	Retrospective cohort	260 neonates, GA < 29 weeks, BW < 1350 g	102 IP-158 evaluated for PDA at 26 h (117 received indomethacin)	0.1 mg/kg/day at <24 h for 3 days (IP) 0.2 mg/kg at 36 h followed by 2 doses, every 12 h 0.1–0.2 mg/kg (PDA)	Ιncidence of sIVH in patients receiving IP versus indomethacin for confirmed PDA.	sIVH: OR 0.27, 95% CI 0.10–0.77	12.5	Reduced incidence of sIVH with IP compared to early echocardiographic strategy	Retrospective, non-randomized Single-center Lower GA in neonates in the IP group Small sample size
Nelin, 2017 [86]	Retrospective cohort	671 outborn neonates, GA < 28 weeks	530 IP-141 control	ND	The effect of IP on mortality and preterm morbidities	IVH any grade: 55% vs. 53%, *p* = 0.63 sIVH: 21% vs. 23%, *p* = 0.64	IVH any grade: 50 sIVH: 50	IP was not associated with lower IVH rates IP was associated with improved survival rates	Retrospective, non-randomized Only neonates transferred to a level IV NICU Prolonged recruitment IP protocols differed in different centers
Gillam-Krakauer, 2021 [82]	Retrospective cohort	384 neonates, GA < 29 weeks	299 IP-85 control	0.2 mg/kg at 12 h (single dose)	The effect of IP on IVH, PDA, and motor function	IVH: OR 0.58, 95% CI 0.36–0.94	IVH any grade: 14.3 sIVH: 50	Decreased IVH rates with IP, in the gestation-adjusted model IP was associated with decreased mortality No increased risk of acute kidney injury	Retrospective, non-randomized Significantly lower GA in the IP group More neonates in the control group were outborn Adherence to the protocol was not mandatory
Clyman, 2022 [87]	Intention-to-treat, cohort-controlled	106 neonates, GA < 25 weeks	68 IP-38 controls	0.2 mg/kg at <24 h, followed by 2–4 doses 0.1 mg/kg	Mortality, incidence of IVH and other preterm morbidities	sIVH: OR 0.94, 95% CI 0.30–2.92	12.5	IP was not associated with a significant reduction in IVH or other prematurity-related morbidities IP was associated with a lower risk of PDA associated morbidities	Single-center, small sample Retrospective, non-randomized Prolonged recruitment
Hanke, 2023 [80]	Observational multicenter cohort	1767 neonates, GA < 26 weeks with AIS	195 IP-1572 controls	0.1 mg/kg/day for up to 3 days	Incidence of IVH	IVH any grade: OR 0.47, 95% CI 0.27–0.79 sIVH: OR 0.24, 95% CI: 0.09–0.61	IVH any grade: 23.8 sIVH: 15.9	Significantly reduced IVH rates in preterm neonates with amniotic infection syndrome	Non-randomized Clinical diagnosis of AIS Selection bias Potential cofounders

RCT: randomized controlled trial; GA: gestational age; BW: birthweight; IP: indomethacin prophylaxis; IVH: intraventricular hemorrhage; sIVH: severe intraventricular hemorrhage; PDA: patent ductus arteriosus; ND: no data; CA: corrected age; AIS: amniotic infection syndrome; OR: odds ratio; CI: confidence interval.

### 3.5. Erythropoietin (EPO)

**Figure 5:**
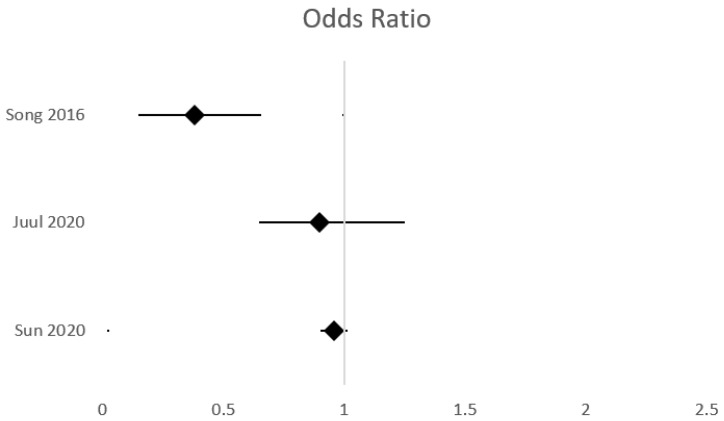
Effect of erythropoietin versus control on severe IVH incidence.

Human erythropoietin (EPO) is a glycoprotein that is primarily known for its role in erythropoiesis, and recombinant EPO (rhEPO) is widely used in preterm neonates to address the issue of anemia of prematurity [111]. Apart from its role as an erythropoietic agent, EPO exerts neuroprotective effects due to its anti-apoptotic, anti-inflammatory, and anti-oxidative properties [112]. Moreover, EPO has been shown to promote cerebral vascular stability through the inhibition of apoptosis and the promotion of angiogenesis in brain capillaries, thereby contributing to the maintenance of cerebral vascular integrity [112,113,114]. A number of studies have investigated the potential neuroprotective effects of rhEPO in neonates at risk of brain injury, including preterm neonates and those with hypoxic–ischemic injury; however, the results regarding its benefit to long-term neurodevelopmental outcomes have been inconsistent [111]. The potential benefits of administering EPO early postnatally, at different dosing regimens with high or low doses, to prevent IVH in preterm neonates have been investigated; however, the data remain inconclusive [111,115] (Table 3, Figure 5).

As less than 2% of EPO crosses the blood–brain barrier, it has been suggested that high doses are necessary to achieve neuroprotective effects [116]. Moreover, studies on experimental animals have shown that the neuroprotective effect of rhEPO is dose-dependent, with high doses being required to improve both short- and long-term outcomes [117,118]. Several studies have been conducted to assess the efficacy and safety of high-dose rhEPO in preterm neonates, based on this evidence. Juul et al. in a multicenter RCT involving 941 preterm neonates with a GA < 28 weeks, compared the rate of short-term morbidities and the neurodevelopmental outcome at the age of two years in neonates that received high-dose rhEPO (1000 U/kg intravenously every 48 h for 6 doses, followed by 400 U/kg subcutaneously three times per week until 32 weeks corrected age) and placebo. The rates of IVH of any grade and sIVH were comparable in both groups (OR 0.90, 95% CI 0.25–1.26), as were the rates of other prematurity complications and neurodevelopmental outcomes [119]. Similar findings regarding IVH (OR 1.0, 95% CI 0.6–1.6) and other complications of prematurity were reported by Fauchère et al. in an RCT, in which preterm neonates with a GA of 26–32 weeks were given three intravenous doses of rhEPO or a placebo at 3, 12–18, and 36–42 h postnatally [120]. Neither of these two large-scale studies identified any safety concerns associated with the administration of high-dose rhEPO, such as an increased risk of premature complications or adverse events, such as arterial hypertension or thromboembolic events, that have been reported in adult populations [119,120]. However, according to the preliminary report of the Erythropoietin for the Repair of Cerebral Injury in Very Preterm Infants (EpoRepair) trial, in which 121 preterm neonates with GA < 32 weeks and moderate or severe IVH were enrolled to investigate the safety and short-term outcomes of high-dose rhEPO (2000 U/kg/day intravenously every 24 h for three days and two further doses at days 10 and 17 after the initial dose), a statistically insignificant increase in the mortality rate was observed in the rhEPO group compared to the placebo group (OR 2.24, 95% CI 0.74–7.66) [121].

Despite the evidence that high doses of rhEPO are required to exert its neuroprotective activity, several studies have shown that the administration of repeated low doses of rhEPO to prevent anemia of prematurity in preterm neonates may result in beneficial neurodevelopmental outcomes. Furthermore, it has been suggested that the cumulative dose of rhEPO, rather than high single doses, is associated with long-term outcomes [122,123]. Song et al. randomized 743 very preterm neonates to receive either 500 U/kg of rhEPO intravenously within the first three days of life and subsequently every other day for two weeks, or a placebo. They reported a significantly lower incidence of sIVH in the EPO-treated group compared to the placebo-treated group (6.6% vs. 15.9%, *p* < 0.001, OR 0.38, 95% CI 0.23–0.62) and significantly lower rates of neurological disability at 18 months [124]. A significantly decreased rate of sIVH in neonates who received the same dosing regimen of rhEPO compared to placebo (OR 0.96, 95% CI 0.94–0.98) was reported in an RCT reanalysis from the same group of authors, involving 1898 very preterm neonates [125]. Moreover, a recent pilot study demonstrated a 97% decrease in the odds of IVH in preterm neonates who received 400 units/kg intravenously three times per week until 32 weeks of corrected age [115]. However, Peltoniemi et al. reported no significant difference in the rate of IVH with the administration of 250 U/kg/day rhEPO in the first 6 postnatal days (OR 0.83, 95% CI 0.15–4.75) [126].

The evidence regarding the efficacy of early postnatal rhEPO in the prevention of IVH remains inconclusive. Ohlsson et al., in a Cochrane systematic review, reported no significant difference in the incidence of IVH of any grade in preterm neonates with a GA of less than 32 weeks who received early postnatal rhEPO (RR:0.98, 95% CI 0.76–1.26). A significant reduction in sIVH was noted (RR:0.60, 95% CI 0.43 to 0.85); however, with a moderate certainty [127]. According to another meta-analysis of 12 studies, a moderate reduction in sIVH was reported with early rhEPO administration, but the certainty of this evidence was low (RR: 0.68; 95% CI: 0.57–0.83) [84].

Several studies have reported no safety concerns regarding the early postnatal administration of rhEPO in preterm neonates; however, further research is required before definitive conclusions can be established [119,120,126,127]. Although concerns were raised in previous versions of the Cochrane review about an increased risk of retinopathy of prematurity (ROP) in preterm neonates treated with rhEPO, a recent Cochrane systematic review concluded with high certainty that there is no increased risk of severe ROP following early postnatal rhEPO administration (RR: 1.24, 95% CI 0.81–1.90) [127].

The current literature on the efficacy of rhEPO in preventing IVH in preterm neonates, whether administered in high or low sustained doses, is inconsistent. Several RCTs have been conducted; however, there are significant variations in the dosing regimens and study populations. Promising results regarding the efficacy and safety of rhEPO have been reported. However, other studies, including a large, robust RCT involving a cohort of extremely preterm neonates with a GA of less than 28 weeks, the most vulnerable population, have failed to demonstrate a benefit regarding IVH prevention [119]. Therefore, in the absence of conclusive evidence, the routine administration of rhEPO in preterm neonates to prevent IVH is not currently recommended.

**Table 3:** Studies investigating the efficacy of early prophylactic erythropoietin administration in the prevention of IVH in preterm neonates.

Author	Type of Study	Population	Study Groups	Intervention	Objectives	Outcome of Interest	Main Conclusions	Limitations
Ohls, 2014 [122]	RCT	99 neonates, BW 500–1250 g	33 rhEPO-33 darbepoetin-33 placebo	400 U/kg rhEPO sc three times per week until 35 weeks PMA	Preterm morbidities Neurodevelopmental outcome at 18–22 months	sIVH (EPO vs. control): 9.4% vs. 23%	No statistically significant difference in the rate of sIVH and other prematurity complications. Fewer transfusions and exposure to fewer donors.	Small sample size Short follow-up period
Fauchere, 2015 [120]	RCT	443 neonates, GA 26–32 weeks	229 rhEPO-214 placebo	3000 U/kg iv rhEPO 3 doses (<3, 12–18, and 36–42 h)	Neonatal morbidities Neurodevelopmental outcome at 24 months	IVH any grade: OR 1.0, 95% CI 0.6–1.6	No adverse effects. No significant differences in prematurity complications.	Short follow-up period
Song, 2016 [124]	RCT	743 neonates, GA < 32 weeks	336 rhEPO-377 placebo	500 U/kg iv rhEPO, initial dose <72 h, every 48 h for 2 weeks	Mortality/neurodevelopmental outcome at 18 months	sIVH: OR 0.38, 95% CI 0.23–0.62	Significantly reduced incidence of sIVH. Better neurodevelopmental outcome.	Limited number of neonates with GA < 28 weeks and BW < 1000 g More males Short follow-up period
Peltoniemi, 2017 [126]	RCT	39 neonates, BW 700–1500 g, GA < 30 weeks	21 rhEPO-18 placebo	250 U/kg/day iv rhEPO for 6 days	Effect of rhEPO administration without iron supplementation in neonatal morbidities and 2 year outcome	IVH any grade: OR 0.83, 95% CI 0.15–4.75	No benefit on IVH incidence or neurodevelopmental outcome at 2 years. No significant difference in other prematurity complications.	Short follow-up period
Juul, 2020 [119]	RCT	941 neonates, GA 24–28 weeks	376 rhEPO, 365 placebo	1000 U/kg iv every 48 h for 6 doses, followed by 400 U/kg sc three times per week until 32 weeks PMA	Neonatal morbidities Neurodevelopmental outcome at 24 months	sIVH: OR 0.90, 95% CI 0.25–1.26	No benefit on neurodevelopmental outcome at 2 years. No significant difference in the rate of prematurity complications.	Short follow-up period
Sun, 2020 [125]	RCTs reanalysis	1898 neonates, GA 24–32 weeks	950 rhEPO, 948 placebo	500 U/kg iv rhEPO, initial dose <72 h, every 48 h for 2 weeks	Effect on ROP and other neonatal morbidities	sIVH: 0.96, 95%CI 0.94–0.98	Significantly lower rates of IVH, NEC and mortality. No significant impact on the incidence of ROP	Limited number of neonates with GA < 28 weeks and BW < 1000 g More males Participants from studies with different objectives
Fernandez, 2025 [115]	Pilot study	40 neonates, GA < 32 weeks	33 rhEPO, 7 placebo	400 U/kg iv three times per week until 32 weeks PMA	Incidence of IVH	IVH any grade: 3 days: 6.5% vs. 71.4%, 10 days: 6% vs. 28.6%	Significantly reduced incidence of IVH.	Small sample study Limited generalized ability

RCT: randomized controlled study; BW: birthweight; rhEPO: recombinant erythropoietin; PMA: postmenstrual age; IVH: intraventricular hemorrhage; sIVH: severe intraventricular hemorrhage; GA: gestational age; ROP: retinopathy of prematurity; NEC: necrotizing enterocolitis.

### 3.6. Insulin-like Growth Factor-1 (IGF-1)

Insulin-like growth factor-1 (IGF-1) is a mitogenic hormone involved in numerous physiological processes, including growth, angiogenesis, and differentiation [128,129]. It is an essential growth factor for central nervous system development as it contributes to the processes of myelination, neurogenesis, and the differentiation of brain cells [130]. Furthermore, it has been demonstrated that IGF-1 through the expression of structural vascular components, promotes vascular maturation and decreases vascular fragility [131,132]. Specific carrier proteins, the IGF-binding proteins (IGFBPs), have been demonstrated to regulate the bioavailability of IGF-1, with approximately 98% of the circulating IGF-1 being bound, and more than 80% of this being bound to IGFBP-3 [133].

During gestation, IGF-1 is a crucial mediator of fetal growth, with levels increasing with increasing gestational age [134]. However, in neonates born preterm, there is a rapid decline in IGF-1 levels following delivery, with levels falling below intrauterine levels, and a slower rise compared to term neonates [135,136]. Low IGF-1 levels in preterm neonates have been associated with poor extrauterine growth, various neonatal morbidities, and neurodevelopmental impairment [128].

Several studies have investigated the potential role of recombinant (rh) IGF-1/IGFBP3 supplementation, intending to achieve a concentration within the normal intrauterine range, reducing the short- and long-term morbidities of preterm birth, including IVH, bronchopulmonary dysplasia (BPD), ROP, and neurodevelopmental impairment [132,136,137,138,139,140]. A number of studies have examined the pharmacokinetic properties of rhIGF-1/IGFBP3 in neonates, a population with unique physiological and developmental characteristics. These studies have concluded that the half-life is much shorter in neonates than in older populations, and that a continuous intravenous infusion of rhIGF-1/IGFBP3 is necessary to achieve serum concentrations comparable to those expected in utero for a specific GA [133,139,140,141]. Following these studies, Chung et al. developed a population pharmacokinetic model, concluding that a continuous intravenous infusion of 250 μg/kg/day would achieve and maintain serum concentrations within the target range [142]. Although adverse effects such as hypoglycemia, sepsis, and intracranial hypertension have been reported in older populations, there have been no reported safety concerns in the neonatal population [133,139,140].

Due to the role of IGF-1 in the process of vascular maturation and its contribution to the maintenance of vascular stability, it has been hypothesized that the administration of rhIGF-1/IGFBP3 could have a beneficial effect in the prevention of IVH in preterm neonates [132,143]. Gram et al. demonstrated the potential role of rhIGF-1/IGFBP3 in preventing IVH by showing that its administration led to the upregulation of gene expression of factors implicated in cerebrovascular maturation, particularly choroid plexus genes, using a preterm pup model [132].

In a phase 2 multicenter RCT, 61 preterm neonates with a GA of 23 to 27^+6^ weeks were randomized to receive rhIGF-1/IGFBP3 at a dose of 250 μg/kg/day as a continuous intravenous infusion from the first postnatal day until the corrected age of 29^+6^ weeks and 60 to standard neonatal care, to evaluate the effect of rhIGF-1/IGFBP3 on the incidence of prematurity complications. The rate of sIVH in the rhIGF-1/IGFBP3-treated population was lower, although statistically insignificant, compared to the controls (13.1% vs. 23.3%, respectively). Among the 24 neonates with >70% of serum IGF-1 concentrations in the target range, the incidence of sIVH was 8.3%. However, as the authors note, an exposure-response relationship between IGF1 levels and the severity of IVH could not be determined due to the small number of events [138]. In a post hoc analysis of this RCT, which included only neonates without pre-existing IVH at the time of enrolment, a trend towards a lower rate of sIVH was reported in the group of neonates treated with rhIGF-1/IGFBP3 compared to those that received standard care (25% vs. 40.5%, respectively). However, the difference was not statistically significant (Table 4) [144].

In conclusion, the existing evidence regarding the potential role of IGF-1 in IVH prevention is encouraging but scarce. The current evidence primarily derives from pharmacokinetic and phase II trials, and it is currently considered an experimental strategy for preventing IVH. Further large-scale, randomized, placebo-controlled trials are needed to provide more conclusive results and support the implementation of this strategy for preventing IVH in clinical practice.

### 3.7. Stem Cells

Stem-cell-based therapies, particularly mesenchymal stem cell (MSC) therapies, are an evolving field of research that shows great promise in the treatment of various types of disease. During the perinatal period, umbilical cord blood and tissue are valuable sources of MSCs, which are characterized by reduced immunogenicity, high differentiation potential, and easy, non-invasive collection [145,146,147].

It has been hypothesized that stem-cell-based interventions, particularly umbilical cord blood, may offer a valuable approach to managing various neonatal morbidities, with a particular focus on prematurity complications. Consequently, several preclinical and early clinical studies have been conducted to investigate the safety and feasibility of these interventions in the neonatal population, as well as their efficacy on various neonatal morbidities [148]. Various dosing regimens and routes of administration have been studied in term and preterm neonates. Intratracheal administration of 1–2 × 10^7^ cells has been studied in two cohorts of nine and 12 extremely preterm neonates on the second postnatal week for bronchopulmonary dysplasia in two phase I open-label dose-escalation studies. No adverse effects were noted [149,150]. Ahn et al. administered intraventricularly up to 1 × 10^6^ cells/kg in nine extremely preterm neonates with sIVH with no reported immediate side effects [151]. In an open-label, phase I trial, Buckhart et al. infused 1–3 × 10^6^ cells into the myocardium of ten infants with hypoplastic left heart syndrome. No treatment-related adverse events were reported [152].The feasibility and safety of autologous and allogenic umbilical cord blood cells administration in cohorts of term neonates with hypoxic–ischemic encephalopathy have been studied. A favorable safety profile was demonstrated in a range of dosing regimens that were studied, ranging from one dose of 6 × 10^6^ cells/kg during the first 72 h to four doses of 1 × 10^5^/kg [153,154,155]. Phase I trials have examined the intravenous administration of umbilical cord blood cells in preterm neonates. Yang et al. administered 5 × 10^7^/kg autologous, volume- and RBC-reduced, non-cryopreserved umbilical cord blood cells to 15 preterm neonates with a GA < 37 weeks [156]. Moreover, in a more recent study, Zhou et al. administered 2.5–5 × 10^7^ autologous cryopreserved umbilical cells to 23 extremely preterm neonates on the second week of life [156]. The two studies reported a favorable safety profile [156,157]. The feasibility of collecting a sufficient quantity of autologous umbilical cord blood from extremely preterm neonates has not been extensively studied. Evidence from two studies indicates that adequate collection in this population is challenging but feasible. The findings of both studies demonstrated that the collection process was successful in over 80% of cases [157,158].

Research is increasingly focusing on the potential of MSCs to prevent and treat multiple neonatal conditions, with a particular attention on perinatal brain injury [148,151,157,159]. It has been demonstrated in vitro that MSCs represent a source of growth factors such as IGF-1, epidermal growth factor, and interleukin 11, which are crucial for oligodendrocyte maturation. In preclinical models of encephalopathy of prematurity, MSCs have been shown to promote myelination and oligodendrocyte maturation and to reduce inflammation [160,161]. A recent meta-analysis of preclinical studies concluded that, while further research is required, umbilical cord blood-derived cells represent a promising intervention for perinatal brain injury [159].

The hypothesis that stem cells may have a role in the prevention of IVH has been proposed based on their known angiogenic properties and the expression of growth factors such as IGF-1 and vascular endothelial growth factor. However, although several studies have investigated the role of MScs in mitigating brain injury following sIVH, to date, there is a paucity of data to support the hypothesis of their role in IVH prevention [151,162]. Kotowski et al. enrolled 20 preterm neonates with GA < 32 weeks who developed anemia and received either an autologous umbilical cord blood transfusion (n = 5) within the first five postnatal days or an allogeneic red blood cell transfusion (n = 15). A significantly reduced incidence of IVH was noted in neonates who received an autologous umbilical cord blood transfusion compared to the control group (*p* = 0.07) [163]. In a phase 2, non-randomized, placebo-controlled trial, Ren et al. administered a single intravenous dose of autologous cord blood mononuclear cells within 8 h after birth to preterm neonates with a GA of less than 35 weeks, to assess whether a decreased rate of prematurity-related complications would be observed. No significant difference in the rate of IVH was reported between the two groups (*p* = 0.962) [164].

A recent Cochrane review concluded that there is currently no evidence to support the use of stem-cell-based interventions in preventing IVH in preterm neonates. Therefore, this approach is not yet applicable in clinical practice. The authors suggest that rigorous preclinical studies are needed to address key questions in order to inform the design of future human studies [162]. The findings from preclinical studies, including the potential benefits of the treatment, the optimal dosing and administration route, the target population, and cell selection and processing, can be used to design human studies to assess safety and optimal dosing at the initial stage. Subsequent to this, the conduction of large RCTs is necessary to ascertain the potential beneficial effects of stem-cell-based interventions in the prevention of IVH and the long-term outcomes.

Based on the promising evidence from preclinical and early studies that stem-cell-based interventions have the potential to mitigate brain injury, there has been an increasing interest in the potential benefits of human milk-derived stem cells. It has been demonstrated that human milk contains pluripotential stem cells, which, in conjunction with bioactive components such as growth factors and cytokines, have the potential to offer protection from brain injury. Furthermore, human milk is readily available, obviating the necessity for pre-planned procedures, processing, and storage [165,166,167]. The intranasal route has been proposed for administration, given its high vascularity and direct connection to the CNS [168].

Following promising preclinical results, several small observational studies have been conducted on preterm neonates to evaluate the potential neuroprotective effects of intranasally administered human milk [168]. Hoban et al. demonstrated that intranasal administration of the mother’s own milk was feasible and well tolerated in a small cohort of 37 neonates with IVH [166]. In a non-randomized pilot study, Keller et al. investigated the potential beneficial effects of intranasally administering human milk to very-low-birth-weight neonates with sIVH. They observed a trend towards lower incidences of porencephalic defects, ventricular dilation, and hydrocephalus in the group of neonates that received human milk intranasally [169]. Gallipoli et al. also reported non-significant differences in outcome in preterm neonates with sIVH who were administered intranasal human milk, although these were more favorable than those of a historical control [170]. Although the potential protective effects of administering human milk intranasally to prevent IVH have not yet been studied, this represents a promising area of research for future preclinical and large-scale clinical studies.

### 3.8. Hemostatic and Anticoagulant Agents

It has been proposed that thrombosis in the cerebral venous system may contribute to the pathophysiology of IVH [171]. Research has indeed demonstrated that neonates with thrombophilia are at an increased risk of intraventricular hemorrhage [172,173]. These observations have led to the evaluation of several factors aimed at balancing coagulation and preventing the formation of blood clots, as a potential preventive strategy for IVH.

Heparin has the function of activating antithrombin, which inactivates coagulation proteases. It has been hypothesized that this could reduce the risk of thrombi formation in the cerebral microvasculature. In an RCT, Birch et al. reported a trend towards reduced IVH progression in the group of neonates who received total parenteral nutrition (TPN) with 0.5 IU/mL heparin, compared to those who received no heparin [174]. However, a former study by Lesko et al. reported that the risk of IVH was fourfold higher in neonates who received heparin [175]. A Cochrane review of two RCTs, involving 155 neonates, reported with very low certainty of evidence, no difference in the incidence of IVH between neonates treated with heparin compared to placebo (RR 0.93, 95% CI 0.61–1.41) [176].

Antithrombin is a modulator of coagulation, given that it represents the primary inhibitor of both thrombin and other coagulation factors. It has been demonstrated that the activity of antithrombin is lower in preterm neonates, particularly in those with sepsis and RDS [177]. It was hypothesized that administering antithrombin could mitigate early postnatal hypercoagulability and reduce the risk of IVH [178]. Brangerberg et al. assessed the IVH rates in a cohort of 103 neonates with a mean GA of 28.9 weeks who received a single dose of antithrombin III at birth. They reported IVH in 13% of this cohort, with no cases of grade IV IVH, which is lower than the epidemiological rate during that period [177]. In a subsequent randomized study, Fulia et al. reported no benefit in terms of the occurrence of IVH with antithrombin administration [179]. A Cochrane review concluded, with a low level of certainty, that no advantage was observed in terms of the incidence of IVH in preterm neonates who received antithrombin compared to those who received a placebo (RR 1.30, CI 95% 0.87–1.93) [178].

Another drug that has been investigated as potentially beneficial in IVH reduction is ethamsylate. It has been shown that ethamsylate increases platelet aggregation and reduces bleeding time [180]. The existing literature is restricted to studies conducted until 1990s, and provides conflicting evidence [181,182]. A Cochrane review reported a significant decline in the IVH rate in preterm neonates with a GA of less than 31 weeks or a birthweight of less than 1500 g who received ethamsylate (RR 0.63, 95%CI 0.47–0.86) [180]. However, the authors note that this should be interpreted with regard to the differences in perinatal care during previous decades. Moreover, no benefit was observed concerning survival or neurodevelopmental outcome [180].

The potential contribution of coagulopathy in preterm infants to the occurrence of IVH has led to the investigation of fresh frozen plasma (FFP) as a preventive strategy for IVH. FFP contains a variety of plasma proteins, including procoagulants and inhibitors of coagulation factors [183,184]. Dani et al. compared the IVH rate between 127 preterm neonates who received FFP following pathological coagulation screening, and 91 neonates who received FFP following bleeding. A decreased incidence of IVH was reported in the screening group (RR 0.65; 95% CI, 0.44–0.98) [184]. A previous RCT also demonstrated beneficial effects [185].

While the aforementioned agents have theoretical rationale, the existing literature is inconclusive and limited by significant methodological issues. Therefore, none of these strategies is recommended for the routine prevention of IVH in preterm neonates.

## 4. Conclusions

IVH is a common complication of prematurity, and despite recent advances in perinatal care, it remains a significant concern in terms of survival and long-term neurodevelopment. Despite the efficacy of several preventive measures that have been implemented in routine clinical practice, such as antenatal corticosteroid administration, the increasing survival of preterm neonates, and particularly of neonates at the border of viability, necessitates the recognition and application of strategies to reduce the incidence of IVH further and improve both survival and the quality of life of these neonates.

The implementation of IVHPB in routine clinical practice, to minimize hemodynamic and cerebral perfusion fluctuations, through strategies including head positioning, minimal handling, stress and pain avoidance, avoidance of blood pressure fluctuations, and rapid intravenous fluid administration and blood withdrawal, represents a promising strategy. Nevertheless, it is imperative that appropriate education is provided and that measures are implemented to ensure adherence to achieve the desired efficacy. There is significant controversy regarding optimal head positioning. Although the tilted midline head position is widely used during the first days of life in preterm neonates, further research is required before it can be recommended universally.

Despite the proposal of several pharmacological agents as potentially useful in the prevention of IVH, controversies persist, and no agent has yet been proven to be efficacious to be introduced in the routine care of preterm neonates. Indomethacin is a drug that has been used for decades to promote ductal closure in neonates. Several large RCTs and observational studies have proven its effectiveness in preventing IVH. However, its efficacy has been disputed in a number of studies, and given the absence of proven longitudinal benefit, its universal administration in neonates is not recommended. Recombinant erythropoietin is a drug used to treat anemia of prematurity. Although the evidence from different studies has been inconsistent, it has shown promising results in preventing IVH. There is very little data on the efficacy of administering rhIGF-1/IHFBP3 to preterm neonates to restore normal intrauterine levels and prevent IVH.

Although the potential role of indomethacin, rhEPO, and rhIGF-1/IGFBP3 in preventing IVH in preterm neonates has been investigated, and encouraging results have been reported, larger prospective studies and RCTs are needed to evaluate their efficacy and provide conclusive evidence and recommendations. Regenerative cell administration is a promising, rapidly evolving domain that has the potential to be useful in the management of preterm neonates. The efficacy of stem-cell-based interventions in the prevention of IVH remains to be investigated.

## Figures and Tables

**Table 4:** Studies investigating the efficacy of IGF-1/IGFBP3 administration in the prevention of IVH in preterm neonates.

Author	Type of study	Population	Study Groups	Intervention	Objectives	Outcome of interest	Main conclusions	Limitations
Ley, 2019 [138]	Multicenter RCT	121 neonates, GA 23–27^+6^	61 intervention, 60 standard care	250 μg/kg/day continuous iv until CA 29^+6^	Incidence of prematurity complications	sIVH: 13.1% vs. 23.3%, *p* > 0.05	Non-significant decrease in sIVH rates Reduction in sBPD	Small proportion of neonates had >70% of serum IGF-1 in the target range Central randomization by GA Inconsistency in adverse events report
Horsch, 2020 [144]	RCT reanalysis	104 neonates, GA 23–27^+6^, no preexisting IVH	52 intervention, 52 standard care	250 μg/kg/day continuous iv until CA 29^+6^	Effects on brain injury	IVH any grade: 25% vs. 40.5%, *p* > 0.05	Although not significant, the beneficial effect on IVH rates was more pronounced in neonates without pre-existing IVH.	Small sample The primary endpoint of the initial study was the incidence of ROP, it was not powered for IVH reduction

IGF-1/IGFBP3: insulin-like growth factor-1/IGF-binding protein 3; RCT: randomized controlled study; GA: gestational age; IVH: intraventricular hemorrhage; sIVH: severe intraventricular hemorrhage; ROP: retinopathy of prematurity; CA: corrected age; sBPD: severe BPD.

## Supplementary Materials

The following supporting information can be downloaded at: https://www.mdpi.com/article/10.3390/jcm14196763/s1, Supplementary Table S1: SANRA checklist.
