# Supplementary material for: Promising Preventive Strategies for Intraventricular Hemorrhage in Preterm Neonates: A Critical Review
Source: J Clin Med. 2025 Sep 24;14(19):6763. doi: 10.3390/jcm14196763 (PMC12524917; doi:10.3390/jcm14196763)
Supplement: Supplementary file 1 [file jcm-14-06763-s001.zip › jcm-3839673-supplementary.pdf]

Supplementary Table S1. SANRA Checklist

Item	Description	Score (0-2)	
1	Justification of the article's importance	2	IVH in preterm neonates is a significant cause of morbidity and mortality. The review presents promising preventive strategies of IVH.
2	Statement of aims	2	The review aims to summarize current evidence on debatable and promising preventive strategies for IVH in preterm neonates.
3	Literature search	2	PubMed, Scopus, Cochrane Library, and Google Scholar were searched using a defined strategy: "("intraventricular hemorrhage" OR "IVH") AND ("preterm neonates" OR "preterm infants") AND ("prevention" OR "prevention bundles" OR "indomethacin" OR "erythropoietin" OR "insulin-like growth factor 1" OR "IGF-1" OR "stem cells")"
4	Referencing	2	References include previous and recent original studies, systematic reviews, and meta-analyses.
5	Scientific reasoning	2	Critical discussion of efficacy, limitations, and gaps in existing literature.
6	Presentation of data	2	Logical structure with sections for introduction, methods, preventive strategies, and conclusion.
